# Anti-diabetics and the Prevention of Dementia: A Systematic Review

**DOI:** 10.7759/cureus.49515

**Published:** 2023-11-27

**Authors:** Ethan Slouha, Fadi Ibrahim, Atbeen Rezazadah, Sarah Esposito, Lucy A Clunes, Theofanis F Kollias

**Affiliations:** 1 Anatomical Sciences, St. George's University School of Medicine, True Blue, GRD; 2 Pharmacology, St. George's University School of Medicine, True Blue, GRD; 3 Pharmacology, St. George's University, St George's, GRD; 4 Microbiology, Immunology and Pharmacology, St. George's University School of Medicine, True Blue, GRD

**Keywords:** dementia, insulin, anti-diabetics, diabete type 2, neurology

## Abstract

Type 2 diabetes mellitus (T2DM) is a worldwide epidemic that is only increasing as the years progress, and as of 2019, affecting over 37 million. T2DM is a chronic condition caused by reduced insulin secretion and increased insulin resistance. Due to insulin not operating at optimal conditions, blood glucose rises and remains high, thus disturbing metabolic hemostasis. Many complications can arise from T2DM, such as coronary vascular disease, kidney damage, eye damage, and, quite significantly, dementia. It is theorized that dementia from T2DM stems from the fact that the brain is susceptible to hyperglycemic conditions, which are promoted by the increase in insulin resistance of target cells in the central nervous system. This directly affects cognitive processes and memory, which correlates to decreased temporal and front lobes volume. The risk of diabetic complications can be minimized with therapeutic interventions such as oral-antidiabetic (OAD) agents and insulin. Several OADs are on the market, but the first-line agent is metformin, a biguanide that decreases glucose production and increases insulin sensitivity. This paper aims to determine if currently prescribed OADs can help slow cognitive decline and reduce the risk and incidence of dementia as a complication of T2DM. Studies found that, for the most part, all OADs except sulfonylureas (SU) significantly slowed the decline of cognitive function and reduced the risk and incidence of dementia. SU’s were shown to increase the risk of dementia in most studies. Of all the OADs, thiazolidinediones may be the most beneficial drug class for reducing the risk of dementia in T2DM patients. Future research should focus on whether early intervention with specific classes of OADs can not only improve glycemic control, leading to decreased hyperglycemia but also prevent the build-up of damaged brain tissue and help to reduce the risk and incidence of dementia in patients with T2DM.

## Introduction and background

Type 2 diabetes mellitus (T2DM)

As of 2019, over 37 million Americans had T2DM, making it one of the most common metabolic disorders globally [1,2]. T2DM is a long-term condition that results in excess glucose circulating in the blood [1]. T2DM stems from two main problems: the pancreas does not produce the optimal amount of insulin, and the receptors of insulin are unable to respond to insulin, leading to significantly higher levels of glucose [1,3]. The pancreas contains beta-cells, which produce and secrete insulin into the blood, which courses through the body until it finds its receptor [3]. When blood glucose is elevated, insulin is released from the pancreas and functions to drop blood glucose via cellular uptake until the blood glucose reaches the optimal level, at which insulin release is decreased [1]. This is disrupted in patients with T2DM as either the beta-cells do not produce insulin or the receptors do not respond, leading to significantly elevated blood glucose levels [1,3].

Organs involved in T2DM include the liver, pancreas, skeletal muscle, brain, small intestines, kidneys, and adipose tissue [3]. In correlation with the organs involved, symptoms include increased thirst, frequent urination, increased hunger, unintended weight loss, slow-healing sores, recurring infections, darkening of the skin on the neck and in the armpits, and numbness/tingling of the hands and feet [1]. Some individuals are more at risk than others, including those with high abdominal fat, being overweight, inactivity, family history, African/African American or Latino, blood lipid levels, age, and polycystic ovary syndrome [1,3]. Being overweight influences the release of insulin due to many inflammatory mechanisms, such as adipokine deregulation and increased free fatty acid release [3]. T2DM also has a high complication rate, including heart and blood vessel disease, nerve damage in limbs, kidney damage, eye damage, slow healing, sleep apnea, and even dementia [1].

T2DM and the link to dementia

T2DM and dementia have reached an epidemic proportion in the USA, and there is a great need to prevent and treat both [4]. T2DM and dementia are very closely linked with the underlying root cause relating to obesity, but there are more similarities most are unaware of [2]. Advanced age is the single greatest risk factor for dementia, with other variables to consider, such as genetics and comorbidities like diabetes playing a role [5]. T2DM significantly predicts cognitive decline and impairment in older adults [4]. T2DM and even prediabetic levels of insulin resistance have been reported to commonly lead to decreased executive function and episodic memory [4]. Some studies have shown that T2DM is associated with a 60% increased risk of dementia; even in recently diagnosed patients, the risk of dementia increased by 16% [4].

The current pathophysiological explanation of dementia is thought to be due to misfolded proteins, which can be caused by cerebrovascular injury, genetic predisposition, or underlying lifestyle habits [5]. The brain is incredibly susceptible to alterations in glycemia, and hyperglycemia induces numerous changes, including changes in osmotic pressure, free radical production from the mitochondria, and neuron apoptosis [6]. With aging, the brain becomes more susceptible to these cellular changes, leading to neuroinflammation and contributing to disease processes such as dementia [6]. This makes sense as the sensitivity of target cells in the central nervous system to insulin is suppressed in T2DM, leading to hyperglycemia and directly affecting memory and other cognitive processes as areas with insulin receptors include the hippocampus, parahippocampal gyrus, thalamus, amygdala, and caudate-putamen shrink [4]. The reduction in brain volume is most notable in the front and temporal lobes [4].

T2DM is uniquely similar to vascular dementia (VD) through micro-vessel damage, in which insulin resistance causes dysfunction in the vascular endothelial cells, thereby leading to vascular compromise within the cerebrovascular system [4]. At the same time, cerebrovascular disease like this is also known to play a large role in the development of Alzheimer’s disease (AD), and VD can co-exist with AD, showing the intricacies that exist [5]. Cardiovascular disease (CVD) causes dysfunctional microglial cells and neuroinflammation, which worsens the development of dementia [5]. And like dementia, CVD is also a complication of T2DM. In 2016, the American T2DM Association published an article referring to AD as “type 3 diabetes” [4]. Studies have shown a 56% increased risk of developing AD if you have T2DM [4]. In a recent genome-wide search, AD and T2DM shared many single nucleotide polymorphisms, suggesting some genetic overlap predisposes individuals to these diseases [4].

Anti-diabetics

T2DM can be treated with OAD that aim to reduce fasting blood glucose and HbA1c. These drugs include metformin, sulfonylurea (SU), thiazolidinediones (TZD), dipeptidyl peptidase-4 (DPP-4i), GLP-1 receptor agonists, sodium-glucose cotransporter 2 (SGLT-2), meglitinides, and alpha-glucosidase inhibitors (aGI). Metformin is the most prescribed first-line anti-diabetic [7]. Metformin functions by causing a reduction in ATP production, leading to an increase in the AMP:ATP ratio, resulting in the activation of AMPK [7]. AMPK activation reduces glucose production by the liver and promotes glucose uptake from the bloodstream into muscle cells [7]. Metformin enhances insulin sensitivity in muscle cells, improving insulin response and facilitating glucose uptake [7]. SU is one of many second-line anti-diabetics that bind to and inhibit K-ATP channels in pancreatic beta cells, altering cell membrane potential, allowing calcium influx, and stimulating insulin secretion [8]. DDP-4i results in rapidly degradation of incretin hormones like GLP-1 [9]. This allows GLIP-1 and GIPD levels to remain elevated in the blood for extended periods after meal consumption, promoting insulin release in response to rising blood glucose [9]. TZD enhances insulin sensitivity by stimulating the peroxisome proliferator-activated receptor gamma (PPARy) and enhancing glucose uptake by skeletal muscle cells through GLUT-4, lowering blood glucose levels, but this requires the presence of sufficient insulin [10].

This is where GLP-1 receptor agonists come into play to augment the physiological reaction of incretin to orally ingested food [11]. SGLT-2 inhibitors function by inhibiting SGLT-2, which is responsible for reabsorbing glucose from the renal proximal tubule, leading to glucosuria [12]. Lastly, aGI, which competitively inhibits the activity of alpha-glucosidase enzymes located in the brush border of enterocytes, inhibits the breakdown of disaccharides and oligosaccharides into monosaccharides, delaying the digestion of carbohydrates [10]. When anti-diabetics become insufficient at controlling T2DM, insulin is brought into the regimen to gain better control of blood glucose levels [13]. This is solely used for severe cases of T2DM, and they come in five primary forms: rapid-acting, short-acting, intermediate-acting, long-acting, and premixed [13-15]. Rapid and short-acting are typically used immediately after meals if blood glucose is uncontrolled, whereas long-acting insulin, also called basal insulin, is usually given in the morning to sustain control for up to 42 hours [13,14]. Anti-diabetics and insulin are crucial to prevent the permanent complications that can occur with uncontrolled T2DM. As mentioned above, a big complication of T2DM is dementia.

Aim

With T2DM and dementia claiming fame as top epidemics globally, finding ways to prevent them becomes crucial. T2DM patient population is significant in size, and most of these patients will develop dementia, possibly early onset dementia. Since studies have indicated that T2DM is a significant risk factor in developing dementia, there may be a way to decrease dementia in those patients, as well as cognitive and executive function. Many anti-diabetic drugs treat T2DM by improving insulin secretion and/or uptake into specific tissues. Because they assist in treating hyperglycemia, these anti-diabetics should be able to slow down the possibility of dementia, worsening cognitive function, and declining executive function in these patients. This paper aimed to compare the different anti-diabetic drugs and the incidence of dementia and their effect on memory and cognitive abilities.

## Review

Methods

A calculated and extensive search of the literature was performed using Science Direct, PubMed, and ProQuest catalogs from January 1, 2013, to December 31, 2023. The search keywords included “Anti-diabetics and dementia,” and “Anti-hyperglycemia and dementia.” The electronic query concentration on peer-reviewed observational and experimental publications on this article’s purpose. Publications that were duplicates, not written in English, and published before 2013 were excluded from the selection process. Once publications were collected, they were evaluated based on their abstracts, titles, full-text accessibility, and study type. The preliminary analysis of the three catalogs resulted in 11,671 publications. Keyword specifiers and the information in the abstract allowed for tailoring the publications down to articles addressing the specific aim of the paper. A total of 52 publications were collected that accomplished this task according to the following criteria.

Inclusion Criteria

The inclusion criteria comprised publications between 2013 and 2003, written in English, conducted on humans, focused on anti-diabetics and dementia, full-text publications, peer-reviewed publications, and observational, case-control, or experimental studies.

Exclusion Criteria

**Figure 1:**
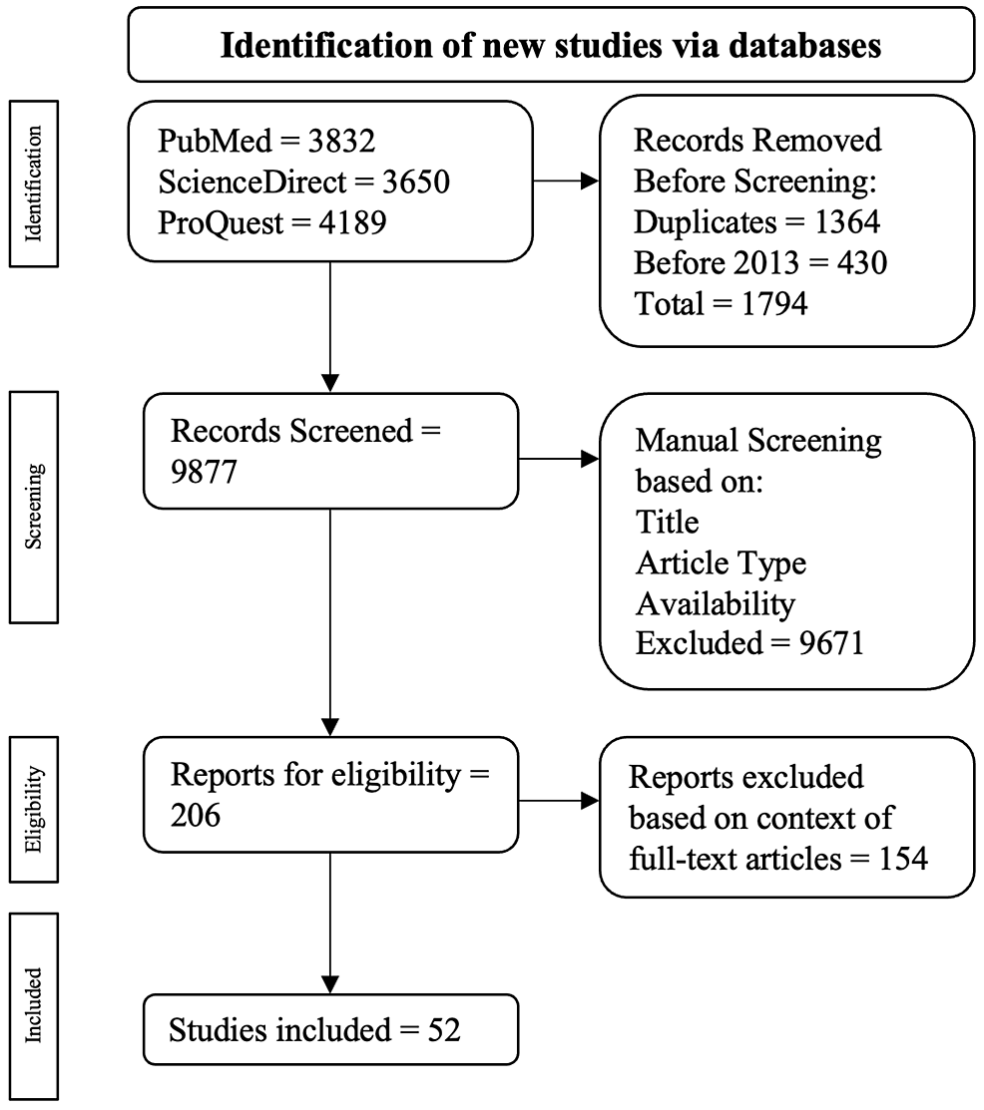
Visual representation of the inclusion and exclusion criteria process. The pathway used for the inclusion and exclusion criteria came from the PRISMA review [16].

The exclusion criteria lead to the exclusion of narrative reviews, case series/reports, and meta-analyses. All duplicates and non-full-text publications were left out as well. This publication's algorithm for inclusion and exclusion criteria is drawn out in Figure 1.

Bias

All publications were reviewed for bias, and it was concluded that the bias in each paper was minimal as all methods were correctly explained. However, some studies grabbed subjects from more extensive studies, which slightly skews the bias. Regarding each article, a moderate GRADE scale score was given. The GRADE (grading of recommendation, development, and evaluation) bias tool was chosen to assess the risk of bias in individual publications, which weighs flaws like publications, indirectness, and imprecision.

Results

A total of 11,671 publications were found: 3,832 were from PubMed, 3,650 were from ScienceDirect, and 4,189. Among the exclusions, 1,364 were duplicate publications, and 430 were published before 2013. This resulted in 1794 publications being excluded during the automatic screening procedure, leading to 9877 publications for manual screening. Publications were manually assessed based on title first, then study type, then abstract and full-text availability, resulting in 206 articles being checked for eligibility by full-text analysis. Ultimately, 52 articles were used.

Most studies found that most anti-diabetics significantly reduced the risk and incidence of dementia in patients with T2DM. Specifically, pioglitazone, a TZD, seemed to be the most successful at reducing the risk and incidence of dementia, especially when paired with metformin. The theory is that pioglitazone provides a significant protective effect because it can cross the blood-brain barrier. Only with TZD was a correlation between dose and effects on the risk and incidence of dementia seen, as metformin was inconclusive between studies. SU was the only drug not associated with preventing dementia, no matter which anti-diabetic it was paired with. They also observed that anti-diabetics, especially metformin, were effective at maintaining a positive effect in preserving cognitive function in immediate and delayed recall.

Discussion

OAD Drugs

It is known that individuals without T2DM have a lower incidence of dementia compared to patients with T2DM [17]. When examining individuals without T2DM, those treated non-pharmacologically for T2DM had a 46% increase in the risk of developing dementia [17]. Comparing pharmacological treatments to non-pharmacological treatments for T2DM, the cognitive status of patients undergoing pharmacology treatment was slightly higher than that of other groups [18]. This is believed to be due to antidiabetics' anti-inflammatory effects on the brain and body [18]. The development of dementia of any type is significantly reduced in patients receiving OAD [19]. Combinations of OAD significantly decrease the incidence of dementia [19]. However, there are many types of OAD, and the relationship between T2DM and dementia changes based on the antidiabetic drug monotherapy [20].

Metformin

Metformin is the first-line OAD for T2DM patients, and its effects on risk and incidence of dementia have been examined. In these studies, the ages of participants ranged from 53.7 to 81 years [21-27]. Most patients had T2DM for at least 5-10 years and were more likely to use insulin and antiplatelets, have depression, smoke, and have heavy alcohol consumption [24, 28]. Common comorbidities included hypertension, hyperlipidemia, CVD, coronary artery disease, heart failure, and anxiety compared to those who did not use metformin [21]. These studies included second-line antibiotics; however, they proceeded with their analysis accounting for these variables to produce metformin-specific results.

Concerning cognition, one study observed that the baseline MMSE was better initially in patients using metformin than those not. Still, the studies were split on the effects of metformin on cognitive decline [22]. Two studies observed that while cognitive impairment was found in 8.3% of patients who used metformin and 14.8% in those who did not, there was no significant change in cognitive assessment, which persisted when accounting for depression [24,29]. However, there was a trend that patients who used metformin for > 6 years were significantly less likely to develop cognitive impairment than those who did not [29]. In agreement, Samaras et al. found that over 6 years, the rate of cognitive decline was significantly less in patients who used metformin, particularly in global cognition and executive function, especially in females. However, there was no significant difference in memory, language, attention/processing speeds, or brain volume [30]. Contrary to both studies, Koo et al. observed that metformin patients tended to have a slightly more rapid deterioration in MMSE scores and were an independent risk factor for this deterioration and Verbal Immediate Recall scores [22].

The disagreement on the effects of metformin continued into the evaluation of all-cause dementia. Two studies observed no significant difference in the risk of dementia between patients who used metformin and those who did not [26,31]. However, four studies observed that metformin may significantly reduce the risk of developing dementia, possibly up to three times lower [30,32-34]. This risk is negatively associated with the dosing of metformin in that the higher the dose, the lower the risk [33]. The actual incident rate of dementia was significantly lower in patients who used metformin than those who did not, and this was seen particularly in men and reduced even further with > 2 years of use [25,33]. Huang et al. observe the opposite in that low doses of metformin reduce the risk of dementia and that there was a positive association between dosing and risk of dementia [21]. Two studies observed that metformin increases the risk of all-cause dementia and agreed with Huang et al. about the positive association between dose and risk of dementia [21,23,35]. Shi et al. found that, while metformin shows a decreased risk of dementia, they did see that the use of metformin <2 years was associated with a significant increase in the risk of dementia [25]. Heneka et al. observed that metformin was found to significantly increase the incidence of dementia [35].

Consistent with cognition and all-cause dementia, AD, as one study observed, there was no clinically significant association between metformin usage and the prevalence of AD [27]. Three studies found that adherence to metformin demonstrated an association with reduced risk of developing dementia regardless of insulin use and that the incidence rates of AD were significantly lower [25,34,36]. Ha et al. determined that the use of metformin was significantly associated with an increased risk of AD development when using metformin >5 years and was independent of depression [37]. Additionally, some evidence suggests a dose-dependent relationship impacting the odds of AD, with only one confirming the relationship in that patients using metformin at higher doses were at great risk of developing AD [27,37].

AD is not the only neurodegenerative disorder that has been evaluated in association with metformin use, as PD seems to be assessed by several studies. The effect of metformin on PD was also split, with one study observing that metformin adherence did not have any impact on the development of Parkinson’s disease [36]. Another study found that more prolonged metformin exposure was associated with a significant decrease in risk and incidence of dementia [25]. Contrary to the previous two studies, Kuan et al. observed a 2.3-fold increased risk of developing PD in patients who used metformin and a trend in increasing the risk with increasing the dose. They also concluded that regarding PD, the cumulative incidence was significantly higher in patients who used metformin [23].

DDP-4 Inhibitors

Second-line antidiabetic drugs are essential to evaluate; most are concurrently used with metformin. DDP-4i is one class of antidiabetics evaluated in this study, and metformin use was also accounted for. When assessing cognition, there seems to be variation in results regarding specific DDP-4i’s, with one study finding no significant difference between linagliptin and placebo when examining accelerated cognitive decline [38]. Concerning sitagliptin, patients receiving sitagliptin had significantly increased MMSE scores compared to baseline [39]. Another study observed that vildagliptin therapy can improve some areas of cognitive function [40].

Evaluating all-cause dementia, DDP-4i’s led to a significant association of decreased risk of dementia, up to 52% in one study, independent of sex, age, and micro- and macrovascular complications [19,41]. Chen et al. observed that therapy durations of DDP-4i exceeding one year presented an increasingly significant protective factor [41]. This is contradicted by Tzeng et al.’s study that showed DDP-4i combined with metformin may increase the risk of dementia [42]. DDP-4is was also found to be associated with significantly lower amyloid-beta burden in the global, lateral parietal, lateral temporal, and anterior/posterior cingulate areas compared to healthy controls [43]. However, Tseng et al. observed the effects of vildagliptin on dementia and observed no significant protective or harmful effect, with the incidence of dementia being similar [44]. They postulated that they may have observed these results because some DDP-4i’s do not cross the blood-brain barrier [44].

Thiazolidinedione

TZDs are another second-line antidiabetic drug usually used in combination with metformin. TZDs, in general, were not evaluated; only pioglitazone and rosiglitazone were studied individually and in comparison, and metformin was adjusted for the analysis. With pioglitazone, it showed a significantly greater five-year dementia-free rate compared to control, with one study observing up to 92.1% of users being dementia-free [35,45]. Overall risk and incidence of dementia were significantly lower in patients who used pioglitazone, ranging from a risk reduction of 23% to up to 90.1% of patients, and was more pronounced at the four-year mark [28,35,45]. It was also observed that there was a dose-response relationship in the risk of reduction of dementia as higher doses further decreased the risk of dementia [28,45]. The decrease in the incidence of dementia did not differ from non-diabetics. Still, pioglitazone may be a protective factor against dementia in patients with T2DM and is time- and dose-dependent [35]. Compared to pioglitazone, rosiglitazone use did not decrease the incidence of dementia, with 2.4% of patients who used rosiglitazone developing dementia compared to 2.5% who did not [46]. Tseng et al. proposed that, like with some DDP-4i, rosiglitazone could not cross the blood-brain barrier, so there is no change in dementia risk or incidence observed [46].

GLP-1 Receptor Agonist

GLP-1 receptor agonists also are prescribed as second-line antidiabetics to treat T2DM, usually in combination with metformin. Three studies observed the effects on cognition and AD using GLP-1 receptor agonists but only focused on liraglutide. Liraglutide was associated with significantly improved performance in cognitive tests such as total learning, MMSE, and animal naming tests, which remained even after adjustment of confounding variables [47]. After adjustment, however, there was no significant difference in changes in short-term memory compared to individuals who underwent lifestyle modifications [48]. There was a significant increase in long-delay-free recall in patients who used liraglutide, which correlated with increased oxyhemoglobin concentration in numerous brain areas as measured on functional-infrared spectroscopy [47]. Concerning AD development, liraglutide displayed significant connectivity in the hippocampi bilaterally, possibly due to liraglutide being able to cross the blood-brain barrier and even improving insulin sensitivity within the brain itself [49].

SGLT-2 Inhibitors

SGLT-2 inhibitors are a second-line antidiabetic also shown to improve cardiovascular complications; however, not many studies have covered the effects on the rate and incidence of dementia. Two studies found that SGLT-2 inhibitor use was associated with a reduced risk and incidence of dementia [49-51]. There was also a lower mortality rate in SGLT-2 inhibitor users with dementia long-term [52].

Insulin

Insulin is the last resort medication when antidiabetics do not work efficiently enough anymore, and it is important to look at its impact on the development of dementia. Insulin usage correlated with a worse health outcome, including a greater decline in global cognitive function [31,53]. This makes sense as some studies have found that using insulin increases the risk of dementia, increasing up to 3x the odds [24,31,32,35,53]. Alkabbani et al. observed that more patients receiving insulin developed dementia compared to those who did not receive insulin, 1% and .71%, respectively. There’s also increased mortality in insulin users who develop dementia [52]. However, it should be mentioned that because this is the last step, dementia would be more likely in those receiving insulin as T2DM has progressed further along. This is supported by Buchman et al. finding that individuals categorized as insulin-dependent diabetics had the greatest risk for dementia [17].

Comparison

This paper aims to highlight the difference in the risk and incidence of dementia between the different OADs. Regarding cognition, metformin overall was found to be associated with the most improvement for immediate and delayed recall compared to other OADs [54]. However, Borzi et al. found that neither metformin nor vildagliptin use, an SGLT-2i, demonstrated a reduction in cognitive performance [55]. DDP-4is, however, were associated with a slower annual MMSE decline compared to patients who used other OADs when comparing weight and imputed analyses [56]. This may be because DDP-4is reduces central and peripheral insulin resistance while indirectly improving cognitive function by reducing oxidative stress and inflammation [55]. With SUs and insulin, they were associated with an increase in MMSE decline compared to DDP-4is [56]. The role of insulin compared to, and along with other OADs, there was a significant increase in MMSE scores and cognitive function after sitagliptin was added to the regimen [39]. However, Seaquist et al. found no strong evidence of a direct and consistent relationship between the use of insulin or rosiglitazone and changes in cognitive performance [57].

Most studies comparing antidiabetics focused on all-cause dementia. When comparing other OADs to TZD, studies observed that TZD was associated with a greater reduction in risk and incidence of dementia [32,58-60]. It should be considered that TZD combinations in Kim et al.'s study consisted of younger patients, which could have contributed to the dementia risk and incidence [58]. Two studies observed that TZD was protective against dementia, especially against aGI and SU groups [60,61]. Compared with SU specifically, it was found that the monotherapy of SU was associated with an increase in the incidence of dementia [61]. TZD was found to be more beneficial in patients < 75 and obese because TZD reduces central obesity, which is also a known risk factor for dementia [61]. One study found that patients using TZD had a greater rate of dementia compared to SUs and metformin, however [62].

Concerning metformin and dementia, some studies found that metformin was superior to SU consistently associated with a decrease in risk and incidence of dementia [34,61,63-65]. Metformin was also found to possibly have a protective effect against dementia, but this was only true in patients < 75 years of age [64,66]. This may be because metformin demonstrated the ability to reduce the expression of innate immune modulators and ApoE levels in cultured human neural cells [63]. Scherrer et al. observed that the use of metformin was significantly associated with a lower risk of dementia only in African-American patients [66].

Regarding DDP-4i, there was a decrease in the risk and incidence of dementia compared to most OADs [67]. However, Wu et al. observed that SGLT-2 may be superior at reducing the risk of dementia compared to DDP-4i’s [51]. SUs were associated with a 9% increase in the risk of dementia, especially glyburide, which was significantly associated with an increased risk of dementia compared to gliclazide [68]. Contrary to this, one study observed that SU was not associated with the risk for dementia [31]. Lastly, insulin therapy only showed an association with a decrease in the incidence of dementia in patients using combined insulin therapy [67]. Bohlken et al. observed an increased risk of dementia compared to Glitazones [32].

The remainder of the studies focused on the links between AD and the use of OADs. TZDs were associated with the biggest reduction in AD, with up to a 56% decreased incidence compared to other OADs, such as alpha-glucosidase [20,60]. This was more true for patients with the ApoE4 gene with a slower decline in immediate recall [54]. However, one study found that when compared to metformin and SUs, TZD was associated with the fastest reduction in immediate recall testing [54]. With metformin, no matter the combination, studies stated a significant decrease in the risk of AD [54,58]. Still, compared to other OADs, metformin was associated with a faster decline in immediate memory over time, specifically in individuals with ApoE4 [54,58]. When focusing more on ApoE4, patients given DDP-4i displayed a greater decline in delayed memory and cognitive function [54]. Kim et al. concurred with the previously mentioned study when they observed that the patients using DDP-4i had a significant decrease in risk of AD compared to those using SU [67]. On the same spectrum, it is found that patients using glipizide, nateglinide, tolbutamide, and glyburide may significantly increase their risk of developing AD [20]. It is essential to mention that despite a lack of research, it was found that metformin, compared to other drugs, led to a significant decrease in both PD and VD [34,58].

One strength that cannot be understated is the diversity in the origins of the studies included in this article. This increases the strength of their results clinically as it is an inadvertent attempt to reference the general population. One limitation of this study was the lack of research focusing solely on SU and dementia, as quite a few studies comparing OADs presented information on SU, but only in comparison. Another limitation of this study is patients' demographics as most of the studies originate from countries in Asia, such as China, South Korea, and Taiwan, which leads to a poor representation of European and Western demographics. There was a good proportion of prospective and retrospective cohorts, which allowed for variations to be analyzed and considered (Table 1) [16].

**Table 1:** Summarizing selected articles covered for this systematic review based on PRISMA protocol T2DM - Type 2 Diabetes Mellitus; AD -Alzheimer's Disease; SU-TZD - Sulfonylurea-Thiazolidinediones; TZD - Thiazolidinediones; VD - Vascular Dementia; GLP-1 - Glucagon-Like Peptide 1; MMSE - Mini Mental Status Exam; DLPFC - Dorsolateral Prefrontal Cortex; HAM-D - Hamilton Depression Rating Scale; SU - Sulfonylurea; PD - Parkinson's Disease; cDDD - Cumulative Defined Daily Dose; DDD - Defined Daily Dose; DPP-4i - Dipeptidyl Peptidase 4 inhibitor; SGL-T2i - Sodium Glucose Cotransporter 2 Inhibitor.

	Author	Country	Design & Study Population	Findings	Conclusion
1	Sluggett et al., 2019 [27]	Finland	Nested case-control (n = 29412)	In this study, the median age was 81 years old, and the average duration of T2DM was 10 years. No association was found between metformin use and the incidence of AD; however, exposure to metformin for >10 years did show reduced odds of AD. Metformin use during only a lag period, as well as low-dose exposure, was seen to have an increased odds of AD.	No association was found between the use of metformin and the incidence of AD. However, dose and duration of use did seem to make an impact as high-dose and long-term usage did have decreased incidence of AD. Initiation of metformin within the three-year lag period consistently had an increased risk for AD.
2	Tang et al., 2022 [61]	USA	Prospective Observational Study (n = 559106)	All-cause dementia had the highest incidence in the SU-TZD combined group and the lowest incidence in the metformin monotherapy group. TZD monotherapy was shown to have the highest mortality rate.	SU monotherapy had a significantly elevated risk of dementia compared to the metformin monotherapy group. TZD monotherapy also had a lower incidence of all-cause dementia. After 2 years, TZD became more protective in maintaining a low incidence of dementia compared to the other groups.
3	Newby et al., 2022 [34]	USA	Comparator study (n = 112,591	It was found that metformin users had a lower risk of all-cause dementia and AD compared to SU users.	Metformin users demonstrated a lower risk of developing all-cause dementia, AD, and VD. This suggests that metformin might offer more neuroprotective effects against dementia.
4	Ng et al., 2014 [29]	China	Prospective study (n = 365)	In the 204 metformin users, it was found to be a significant linear trend of association between the duration of metformin use and cognitive impairment. Moreover, a longer duration of metformin use, > 6 years, was particularly associated with a lower risk of cognitive impairment.	Long-term treatment with metformin among individuals with T2DM could potentially reduce the risk of cognitive decline.
5	Li et al., 2021 [47]	China	Prospective study (n = 50)	After 12 weeks of treatment, the GLP-1 group had significantly improved cognitive performance measured by total learning, animal naming, and MMSE scores. Liraglutide also significantly activated brain parts associated with improved cognitive performance, including DLPFC.	After 12 weeks, liraglutide provided significantly significant improvement in cognitive functioning, including MMSE scores
6	Ha et al., 2021 [37]	Korea	Case-control study (n = 10050)	The use of metformin was associated with an increased chance of developing AD. AD risk was increased in patients who have a longer duration of T2DM. The risk of AD was significantly increased in T2DM patients who had depression.	Metformin was associated with an increased risk of AD, for which depression was additive.
7	Nolasco-Rosales et al., 2023 [24]	Mexico	Cross-sectional study (n =431)	According to the HAM-D, there was no significant association between metformin treatment and cognitive impairment or depression.	This study found no significant association between metformin treatment, cognitive impairment, and signs of depression in patients with T2DM.
8	Orkaby et al., 2017 [64]	USA	Retrospective cohort study (n = 432,922)	The risk for dementia was lower in metformin users compared to that of SU users, especially for those aged 75 and below. A lower dementia risk associated with metformin use for younger veterans of well-controlled HbA1c <7%, good renal function, and who were white.	Among older adults with T2DM, metformin use might be associated with a lower risk of developing dementia compared to the use of SUs, especially in veterans under the age of 75.
9	Lu et al., 2018 [59]	Taiwan	Retrospective cohort study (n = 51,415)	Metformin + pioglitazone had a significantly reduced risk of developing dementia compared to those taking metformin + SU. There was also a decreased risk of developing dementia with the just of pioglitazone alone. Pioglitazone was also more successful in reducing dementia compared to rosiglitazone when both were paired with metformin.	As a second-line treatment in combination with metformin, pioglitazone may provide a protective effect concerning dementia risk.
10	Chou et al., 2017 [45]	Taiwan	Cohort study (n = 19203)	Individuals receiving pioglitazone treatment demonstrated a 23% reduction in risk of developing dementia throughout a 5-year follow-up period. Moreover, this study found that the protective factors associated with pioglitazone were dependent on both the dose and duration of use, with long-term, increased dosing demonstrating a significantly reduced risk of dementia.	Pioglitazone use in patients with T2DM may have an association with a reduced risk of developing dementia. However, the protective effects are influenced most by both dose and duration of treatment.
11	Tseng, 2019 [46]	Taiwan	Retrospective cohort study (n = 5048)	When participants were followed for risk of dementia, it was found that rosiglitazone has no impact on the development of dementia. When specifically compared to metformin, there was a non-significant effect on dementia with rosiglitazone usage.	Rosiglitazone does not increase or decrease the risk of dementia, especially when compared to metformin.
12	Tseng, 2021 [44]	Taiwan	Retrospective cohort study (n = 398806)	In both the matched and unmatched cohorts, vildagliptin had a neutral effect on the incidence of dementia. Neither the cumulative dose nor the cumulative duration of medication usage made any significant changes in the outcomes for dementia. When broken down by time-frames, there was still a null association between vildagliptin and dementia.	Vildaglipitin neither increased nor decreased the risk of dementia, regardless of the dose.
13	Ha et al., 2023 [28]	South Korea	Retrospective Cohort (n = 91218)	Pioglitazone was significantly associated with a reduction in the risk of dementia and was greater among patients with a history of stroke or ischemic heart disease before T2DM onset. Stroke incidence was also reduced by pioglitazone.	Pioglitazone has been shown to reduce dementia and stroke risk.
14	Heneka et al., 2015 [35]	Germany	Prospective Cohort Study (n = 145,928)	Pioglitazone long-term use was significantly associated with a decrease in dementia incidence by 47%. If used for <8 quarters, the risk of dementia was comparable to patients who are nondiabetics, and diabetics without treatment have a 23% increase in risk of dementia.	Pioglitazone has been found to significantly reduce the risk of dementia and decrease the incidence.
15	Vadini et al., 2020 [48]	Italy	Longitudinal, randomized parallel arm study (n = 40)	Findings showed that liraglutide might slow cognitive decline in obese patients treated during the preclinical phase of T2DM	After having similar results regarding weight loss, glycemic control, and insulin sensitivity, results indicated that the liraglutide-treated group had significant increases in short-term memory and a memory composite z-score.
16	Cheng et al., 2014 [62]	Taiwan	Longitudinal Study (n = 67,731)	Participants treated solely with TZD demonstrated a significantly greater risk of developing dementia compared to individuals receiving metformin and SUs, who demonstrated a relatively lower risk of developing dementia.	This study suggests individuals who developed late-life T2DM demonstrated an increased risk of dementia as well. However, this risk was significantly greater in those who solely depended on thiazolidinediones compared to metformin and SUs.
17	Chen et al., 2023 [36]	Taiwan	Retrospective cohort study (n = 31384)	Adherence to metformin demonstrated a significant reduction in the risk of developing dementia. This data was consistent across both male and female patients, ages >65 or ≤ 65 years, and those with or without concurrent insulin treatment. Metformin adherence had no significant association with the development of PD.	Adherence to metformin shows a reduction in the risk of developing dementia, irrespective of the patient's gender, age, or concurrent insulin treatment.
18	Huang et al., 2023 [21]	Taiwan	Prospective cohort study (n = 736473)	Use of metformin at cDDD < 300 and >25 DDD/month was not associated with an incident of dementia. However, those with low intensity had a reduced risk of dementia.	High-dose metformin was not associated with dementia incidence, but low-dose use showed a reduced risk of dementia.
19	Salas et al., 2022 [26]	USA	Retrospective cohort study (n = 12,178)	There is no significant difference associated with starting metformin treatment, as compared to not starting or delaying metformin treatment and the risk of incident dementia.	Early metformin treatment is not associated with significantly reduced development or prevention of cognitive decline.
20	Watson et al., 2019 [49]	USA	Experimental study (n = 43)	At baseline, higher fasting glucose was shown to have decreased connectivity between the bilateral hippocampi and anterior medial frontal structures. After 12 weeks of liraglutide, there was a significant improvement in connectivity compared to the placebo.	Liraglutide was shown to improve brain connectivity, reducing dementia.
21	Chen et al., 2020 [41]	Taiwan	Longitudinal study (n = 66,333)	This study showed that the use of DPP-4i was associated with a reduction in the risk of developing dementia. The protective effects of DPP-4i demonstrated a cumulative pattern, with the best results observed in patients with therapy durations exceeding 1 year.	This paper suggests that the use of DPP-4i is associated with a 21% reduction in the risk of developing dementia in older patients with T2DM.
22	Alkabbani et al., 2023 [53]	Canada	Comparative analysis (n = 33,093)	It was determined that there was an increased risk of developing dementia for insulin users.	There is no significant association in the risk of developing dementia in T2DM individuals who are using insulin.
23	Bohlken et al., 2018 [32]	France	Case-control, retrospective study (n = 16,552)	This paper determined that several factors were associated with alterations in the risk of developing dementia in patients with T2DM. The usage of glitazones, metformin, and SUs showed a decreased risk in comparison to insulin usage, which showed an increased risk. Moreover, higher HbA1c levels are also shown to be an additional risk factor for the development of dementia.	The usage of glitazones, metformin, and SUs are associated with a reduction in the risk of developing dementia. In contrast, the usage of insulin, as well as increased levels of HbA1c, are associated with an increased risk of developing dementia.
24	Samaras et al., 2020 [30]	Australia	Prospective observational (n = 1,037)	Participants using metformin showed a significantly slower decline in global cognitive function and executive function compared to those not on metformin. In addition, it was determined that the incidence of dementia was significantly lower in the metformin group compared to the non-metformin group, suggesting that metformin has cognitive protective properties.	The study suggested that older individuals with T2DM who are receiving metformin experience slower cognitive decline and lower risk of dementia compared to those not taking metformin. Such results suggest a potential protective effect of metformin on cognitive function in older populations with T2DM.
25	Scherrer et al., 2019 [67]	USA	Retrospective cohort study (n = 86,051)	The study found that initiating metformin treatment was associated with a significantly lower risk of developing dementia in the VHA and KPW patient groups. In addition, there was no significant association between dementia risk among those aged 75 years or older and metformin use.	Metformin may have a protective effect against the development of dementia when used as the first-line treatment for T2DM in patients aged 50-70 years. The protective effect was not significant in patients >75.
26	Isik et al., 2017 [39]	Turkey	Observational study (n = 205)	The amount of patients requiring reduced insulin dose was significantly increased in patients on sitalipitin. There was also a significant increase in MMSE in patients who received sitagliptin therapy.	Sitagliptin therapy proved to be more efficacious than metformin in reducing cognitive impairment in T2DM patients.
27	Scherrer et al., 2019 [65]	USA	Comparator cohort study (n = 73,761)	Metformin initiation was associated with a significantly lower risk of dementia in African American patients but no in white patients. The stronger protective association between metformin use and dementia was observed in younger African American patients aged 50-64. No significant dementia risk reduction was found in metformin in patients >75 years old.	Initiating metformin therapy, rather than SU, is linked to a notably lower risk of dementia in younger African-American patients with T2DM, suggesting a potential protective strategy for reducing dementia risk in this specific population.
28	Zhao et al., 2023 [60]	China	Retrospective cohort study (n = 82554)	Approximately 196 out of 100,000 persons developed dementia in the alpha-glucosidase inhibitor group, while ~78 out of 100,000 developed it in the TZD group.	It was determined that TZD usage provided a protective factor against dementia development.
29	Koo et al., 2019 [22]	South Korea	Prospective cohort study (n = 732)	Metformin was not associated with changes in daily living index. There was a rapid deterioration of the MMSE and verbal immediate recall scores in patients taking metformin.	Metformin was associated with a cognitive decline in diabetic patients.
30	Wu et al., 2021 [18]	China	Case-control study (n = 13,691)	In patients involved in the study, patients with treated T2DM performed better with mental intactness compared to patients who were T2DM-free; this statistically significant finding was only pertinent in older individuals rather than middle-aged individuals. Covariables such as depression, smoking, drinking, and an abnormal BMI may affect these findings.	Older patients with treated T2DM had increased performance in terms of mental intactness than T2DM-free patients. This was not seen when examining middle-aged patients.
31	Wu et al., 2020 [54]	Canada	Case-control study (n = 1999)	In patients with normal cognition, metformin was associated with improved memory performance over time. In patients with AD, DPP-4i was associated with a delayed rate of memory decline.	With patients defined as having normal cognition, metformin was associated with better test performance over time, whereas DPP4 inhibitors were associated with lower cognitive decline in patients with Alzheimer’s Disease.
32	Jeong et al., 2021 [43]	South Korea	Retrospective cohort study (n = 282)	AD-related cognitive impairment T2DM patients who were treated with DPP-4is showed to have lower global and temporoparietal amyloid burden than those both without treatment and non-T2DM. Patients treated with DPP-4i have shown a significantly slower longitudinal decrease in MMSE scores.	DDP-4i has been found to have protective effects in AD-related cognitive impairment as results were significantly better than patients without treatment and who did not have T2DM.
33	Seaquist et al., 2013 [57]	USA	Randomized study (n = 10,251)	There was no significant relationship between the use of Rosiglitazone and Digit Symbol Substitution Test (DSST) performance at baseline, suggesting that Rosiglitazone use was not linked to cognitive performance initially. However, among those who aimed for lower HbA1c levels, rosiglitazone was linked to a more significant decline in cognitive performance over time.	Exposure to TZD (like rosiglitazone) might lead to cognitive decline in certain individuals with T2DM, especially those who are pursuing intensive glycemic control. The study also acknowledges that these results could be influenced by factors not accounted for, indicating the need for further investigation.
34	Kim et al., 2020 [33]	South Korea	Cohort study (n = 278,290)	20.3% of all patients developed dementia, and T2DM was associated with dementia at a 1.69-fold increased risk. The risk of dementia was decreased in patients who took oral diabetics when compared to no medicine.	Oral diabetics may be preventative but do decrease the risk of dementia in patients with T2DM.
35	Secnik et al., 2021 [56]	Sweden	Prospective open-cohort study (n = 1,873)	Patients using metformin and DDP-4i experienced a slower cognitive decline over time compared to non-users of these drugs. In contrast, patients using insulin and SUs showed a larger point-wise decrement in MMSE score with annual intervals compared to DDP-4i users.	In patients with T2DM and dementia, the use of metformin and DDP-4i was associated with a slower decline in MMSE scores, suggesting potential cognitive benefits. However, the use of insulin and SUs was associated with larger cognitive declines over time compared to DDP-4i.
36	Secnik et al., 2022 [52]	Sweden	Prospective open-cohort study (n = 132,402)	Among individuals using insulin, both those with dementia and dementia-free subjects had an increased risk of mortality compared to non-users. SU was associated with higher mortality, but this effect was observed only in individuals with dementia. GLP-1a users had a lower risk of mortality compared to non-users in both dementia and dementia-free groups. SGLT2i users had a lower risk of mortality in the dementia and dementia-free groups.	The impact of glucose-lowering drugs on mortality varies depending on dementia status and emphasizes the importance of considering the choice of medication carefully, especially in patients with dementia, to minimize mortality risk.
37	Charpignon et al., 2022 [63]	United states	Cohort study (n = 13,191)	This paper focused on diabetic patients over 50, starting either metformin or SU monotherapy. It was determined that the usage of metformin reduced all-cause mortality as well as the onset of dementia when compared to SUs. Moreover, these benefits are more profound in younger populations.	This study revealed that metformin, when compared to SUs, consistently showed a reduced risk of dementia onset as well as overall mortality in individuals with T2DM, more specifically, those 70 or younger.
38	Bulut et al., 2020 [40]	Turkey	Retrospective, longitudinal study (n = 130)	This study determined that vildagliptin therapy potentially improved certain aspects of cognitive function. In addition, it was also determined that vildagliptin therapy was associated with an improvement in weight as well as metabolic control.	Although requiring further research, this study suggests that therapy with vildagliptin may have the potential to improve both cognitive functions as well as metabolic control in older patients with T2DM.
39	Buchmann et al., 2019 [17]	Germany	Longitudinal random sample (n = 250,000)	It was determined that T2DM was associated with an increased risk of dementia, with individuals who were insulin-dependent showing the highest risk, Moreover, it was determined that the risk of developing dementia increased in obese patients with insulin dependence in comparison to non-obese insulin-dependent patients.	It was further determined that treatment of T2DM played a significant role in the risk of developing dementia, where oral anti-diabetic medication showed the lowest risk, followed by non-pharmacologically treated and insulin-dependent patients. Obesity only played a significant role in the insulin-dependent diabetic population.
40	Borzi et al., 2019 [55]	Italy	Retrospective study (n = 60)	This study concluded that individuals treated with both metformin and vildagliptin did not have any decline in their cognitive performance over the study period. However, this was not the case for individuals treated with metformin monotherapy.	It was determined that combined therapy with metformin and vildagliptin demonstrated a preservation in cognitive function in diabetic function compared to metformin monotherapy.
41	Biessels et al., 2019 [38]	Netherland	Randomized trial (n = 1545)	This paper concluded that linagliptin did not significantly affect the risk of cognitive decline in individuals with T2DM over a 2.5 year period.	In individuals with T2DM using linagliptin. It was determined that there was no modulation of cognitive decline over the 2.5-year period.
42	Akimoto et al., 2020 [20]	United States	Observational study (n = 66,085)	With a focus on patients 65 and older receiving various monotherapies for T2DM, it was determined that, compared to metformin, glyburide showed association with an increased risk of AD. In contrast, rosiglitazone, exenatide, sitagliptin, liraglutide and dulaglutide demonstrated a significantly lower risk of developing Alzheimer’s disease.	With a focus on determining if specific antidiabetic medication could predict the risk of Alzheimer’s disease in patients with T2DM mellitus, it was determined the GLP-1 analogs and rosiglitazone demonstrated a reduction in risk of developing AD.
43	Kuan et al., 2017 [23]	Taiwan	Prospective cohort study (n = 9,302)	Patients receiving metformin demonstrated an increased risk of PD, all-cause dementia, Alzheimer’s disease, and VD.	Metformin increases the risk of developing AD, PD, all-cause dementia, and VD.
44	Shi et al., 2019 [25]	USA	Retrospective longitudinal cohort study (n = 5,528)	The study found that the incidence rate of neurodegenerative disease was significantly lower among patients who received metformin treatment compared to those who did not receive metformin. Specifically, patients with 2-4 years of metformin exposure had a significantly reduced risk of neurodegenerative disease compared to non-users. The risk reduction was even more pronounced in individuals with >4 years of metformin exposure.	Long-term metformin therapy, particularly for more than 2 years, was associated with a lower incidence of neurodegenerative disease in elderly veterans with T2DM.
45	Siao et al., 2022 [50]	Taiwan	Retrospective case-control cohort study (n = 976,972)	The results of this study indicated that the SGLT2i group was associated with a significantly lower risk of incident dementia. This suggests an 11% lower risk of developing dementia in patients using SGLT2i compared to those not using them. Also, the study found that diabetic complications were significantly lower in the SGLT2i group compared to the non-SGLT2i group.	Applicable to real-world practice, patients with T2DM who were prescribed SGLT2i had a lower risk of developing incident dementia compared to those not prescribed SGLT2i.
46	Kim et al., 2020 [33]	South Korea	Prospective cohort study (n = 73,718)	The overall dementia incident rates was 11.3%. Metformin users had a decreased risk in dementia with women being more likely to be protected from dementia	Metformin was round to reduce the risk of dementia.
47	Kim et al., 2021 [58]	South Korea	Retrospective cohort study (n = 701,193)	All-cause dementia and vascular dementia was significantly associated with dual therapy with DPP-4i + metformin, TZD + metformin, and SU + TZD, compared to metformin + SU. Metformin + TZD and metformin + DPP-4i were significantly associated with a lower risk of AD compared to metformin + SU. Compared to those not on TZD, dual therapy for TZD was significantly associated with a lower risk of all-cause dementia, AD, and vascular dementia.	The addition of DPP-4i and TZD as second-line antidiabetic treatment compared to SU may be more beneficial to prevent or slow down dementia.
48	Wu et al., 2023 [68]	Canada	Population-based cohort study (n = 144,839)	SUs were associated with an increased risk of dementia compared to DDP-4i’s. Within the SU’s, Glyburide was associated with an increased risk of dementia compared to gliclazide.	SUs were associated with an increased risk in dementia compared to DPP-4is with a higher risk in Glyburide specifically
49	Kim et al., 2018 [67]	South Korea	Retrospective cohort study (n = 15,104)	The all-cause dementia risk was significantly lower in patients receiving DDP-4i compared to SU, and slightly lower risk of vascular dementia.	DDP-4i might be a better option at preventing dementia compared to SU.
50	Wu et al., 2023 [51]	Canada	Retrospective cohort study (n = 106,903)	Compared to DPP-4i, SGLT-2 inhibitors were associated with a significant decrease risk of dementia, which dapagliflozin showing the lowest risk.	SGLT-2 inhibitors were shown to be more effective at reducing dementia compared to DPP-4i.
51	Weinstein et al., 2019 [31]	USA	Cohort study (n = 3315)	The was an association between insulin use and the increased risk of dementia, and a significant decline in global cognitive function. Metformin or SU did not have an association with brain function and structure.	Insulin leads to an increase risk of demention with a decline in global cognitive function, where as metformin and SU had no associaton with brain function and structure.
52	Tzeng et al., 2023 [42]	Taiwan	Retrospective cohort study (n = 67,281)	Treatments combining DPP-4i and metformin increase the risk of dementia compared to those using just metformin.	DPP-4i may increase the risk of dementia when combined with metformin.

## Conclusions

T2DM is a chronic medical condition characterized by elevated glucose levels in the blood. This condition occurs when the body does not respond to insulin effectively, which can have many serious health consequences, such as cardiovascular and neurological complications. One consequence is the increased risk of dementia. Dementia is a broad term for cognitive symptoms and impairments that affect thinking, memory, and ability to perform daily activities. T2DM can, therefore, lead to specific types of dementia, such as AD and PD, congruent with the numerous etiologies of each dementia.

Metformin, TZDs, DDP-4i, and possibly SGLT-2 inhibitors significantly reduced the risk and incidence of dementia in patients with T2DM. Metformin appeared to be particularly effective in maintaining a positive effect on immediate and delayed recall, which could contribute to preserving cognitive function. TZD specifically showed the greatest reduction, however, in the risk and incidence of dementia, pioglitazone, which can cross the blood-brain barrier and is dose-dependent. It’s important to note that the results concerning second-line antidiabetics involved concurrent use of metformin and other second-line antidiabetics. Insulin, however, has been shown to increase the risk and incidence of dementia. This, though, could be due to the advanced stage of T2DM patients when prescribed insulin.

This extensive analysis of the relationship between OADs and whether they can slow the risk of dementia reveals a complex landscape with varying results. It highlights that specific OADs, such as metformin, TZDs, DDP-4i, and SGLT-2 inhibitors, may offer protective cognitive effects for patients with T2DM but with different success rates. Therefore, understanding the impact of OADs on dementia risk is crucial, given the high prevalence of T2DM and the potential implications for cognitive health. This comprehensive systematic review provides valuable insight into the complex relationship between OAD drugs and dementia. Since dementia develops years before a diagnosis, this review highlights the importance of personalized treatment strategies and early intervention in managing T2DM, considering both cognitive health and glycemic control and the potential benefits of certain OADs in preserving cognitive function in T2DM patients. Further research must confirm these findings and refine treatment recommendations, especially in diverse populations.
